# Transcriptomic analysis of human breast cancer cells reveals differentially expressed genes and related cellular functions and pathways in response to gold nanorods

**DOI:** 10.1007/s41048-015-0005-0

**Published:** 2015-08-21

**Authors:** Teng Zhou, Yipeng Du, Taotao Wei

**Affiliations:** National Laboratory of Biomacromolecules, Institute of Biophysics, Chinese Academy of Sciences, Beijing, 100101 China

**Keywords:** Breast cancer cell, Gold nanorod, RNA-seq technology, Glycolysis, Regulation of actin cytoskeleton

## Abstract

Breast cancer is the leading cause of cancer deaths in women. Recent advances in nanomedicine have shown that gold nanorods (AuNRs), as multifunctional drug delivery and photothermal therapeutic agents, have potential for use in cancer therapy. However, the effect of AuNRs on the transcriptome of breast cancer cells is unknown. In the present study, cells of the triple-negative human breast cancer cell line MDA-MB-231, which has high metastatic activity, were treated with AuNRs for transcriptomic analysis using RNA-seq technology. In total, 3126 genes were found to be up-regulated and 3558 genes were found to be down-regulated in AuNR-treated MDA-MB-231 cells. These differentially expressed genes presumably take part in multiple biological pathways, including glycolysis and regulation of the actin cytoskeleton, and impact a variety of cellular functions, including chemoattractant activity. The distinct gene expression profile of MDA-MB-231 cells treated with AuNRs provides a foundation for further screening and validation of important genes involved in the interaction between AuNRs and MDA-MB-231 cells.

## Introduction

Breast cancer, a type of cancer originating from breast tissue, is the most common cancer in women and is the second leading cause of cancer death in women (Kreiter et al. 2014). Breast cancer can also occur in men, who tend to have poorer outcomes due to delays in diagnosis (Block and Muradali 2013). Prognosis and survival rates for breast cancer vary greatly depending on the cancer type, stage, treatment, and geographical location of the patient. Determining the presence of estrogen and progesterone receptors in breast cancer cells is important for guiding hormone therapy (Lavrovskaya et al. 2005). In addition to hormone receptors, other cell surface proteins, such as HER2, may affect prognosis and treatment. Patients who test positive for HER2 may be treated with trastuzumab (Herceptin), a monoclonal antibody that targets HER2 and significantly improves patient prognosis (Mariani et al. 2009). Cancers that test negative for these hormone receptors and for HER2 are not able to respond to hormone therapy or monoclonal antibody therapy, and patients with those cancers have a lower chance of survival (Kim et al. 2014).

Currently, researchers are pursuing the development of new tools and therapeutic strategies to provide more effective therapies against cancers including triple-negative breast cancers. Recent advances in nanomedicine, a multidisciplinary field that involves the application of nanomaterials in medicine and healthcare, offer a promising alternative for cancer treatment (Farrell et al. 2010; Alexis et al. 2010; Sahoo et al. 2007). Due to their small size and the fact that they can be easily modified, nanomaterials demonstrate significant advantages in gaining access to cancer cells, by either passive or active targeting. In addition, based on their unique intrinsic characteristics and controlled functional properties, nanomaterials have the potential for broad application in the detection and imaging of cancers and in cancer therapy (Tiwari 2012; Bae et al. 2011; Zhang et al. 2008). Among these nanomaterials, gold nanorods (AuNRs) have been widely examined for applications in bioimaging, biosensing, drug delivery, and photothermal therapy due to their unique physiochemical and optical properties (Wang et al. 2014; Vigderman et al. 2012; Jokerst et al. 2012; Alkilany et al. 2012; Cobley et al. 2011). To date, many studies have been performed on the cytotoxicity, biocompatibility, and biodistribution of AuNRs with different sizes, shapes, and surface modifications (Zhang et al. 2013; Albanese et al. 2012; Grabinski et al. 2011; Qiu et al. 2010). Wang et al. investigated the interaction between specific proteins and AuNRs and determined the binding structure of the protein corona on AuNRs (Wang et al. 2013). Zhang et al. have suggested that AuNR exposure causes significant changes to the metabolome in human alveolar epithelial carcinoma cells (Zhang et al. 2013). So far, little is known regarding the effect of AuNRs on the transcriptome of cancer cells.

The recent developments of next-generation sequencing (NGS) allow us to sequence DNA and RNA with higher sample throughput (Siu et al. 2011). Transcriptomic analysis, which studies all of the RNA transcripts under a particular physiological condition, has played a central role in detecting gene expression and transcriptional regulation (Maher et al. 2009). RNA-seq technology, which uses deep-sequencing technologies to directly determine cDNA sequences, is an excellent approach for transcriptome profiling (Costa et al. 2010). Because of its relatively lower price, higher base coverage, and greater precision with measuring transcripts compared with hybridization-based microarray assays, RNA-seq is particularly suited for the detection of transcripts, which correspond to existing genomic sequences (Wang et al. 2009). Curtis et al. have recently revealed novel breast cancer subgroups and their molecular drivers through genomic and transcriptomic analysis (Curtis et al. 2012). However, the transcriptomic changes of breast cancer cells treated with AuNRs have not been investigated until now.

In this study, we used RNA-seq technology to investigate the mRNA transcript changes in MDA-MB-231 human breast cancer cells treated with AuNRs. The uptake of AuNRs into MDA-MB-231 cells was observed using two-photon laser confocal microscopy. Total mRNA samples from AuNR-treated and untreated MDA-MB-231 cells were extracted, and a cDNA library for each sample was constructed. After Illumina HiSeq™ 2000 sequencing, comparative transcriptome analysis was performed to reveal differentially expressed genes and related cellular functions and pathways in AuNR-treated MDA-MB-231 cells.

## Results and discussion

### Uptake of AuNRs into MDA-MB-231 human breast cancer cells

**Fig. 1:**
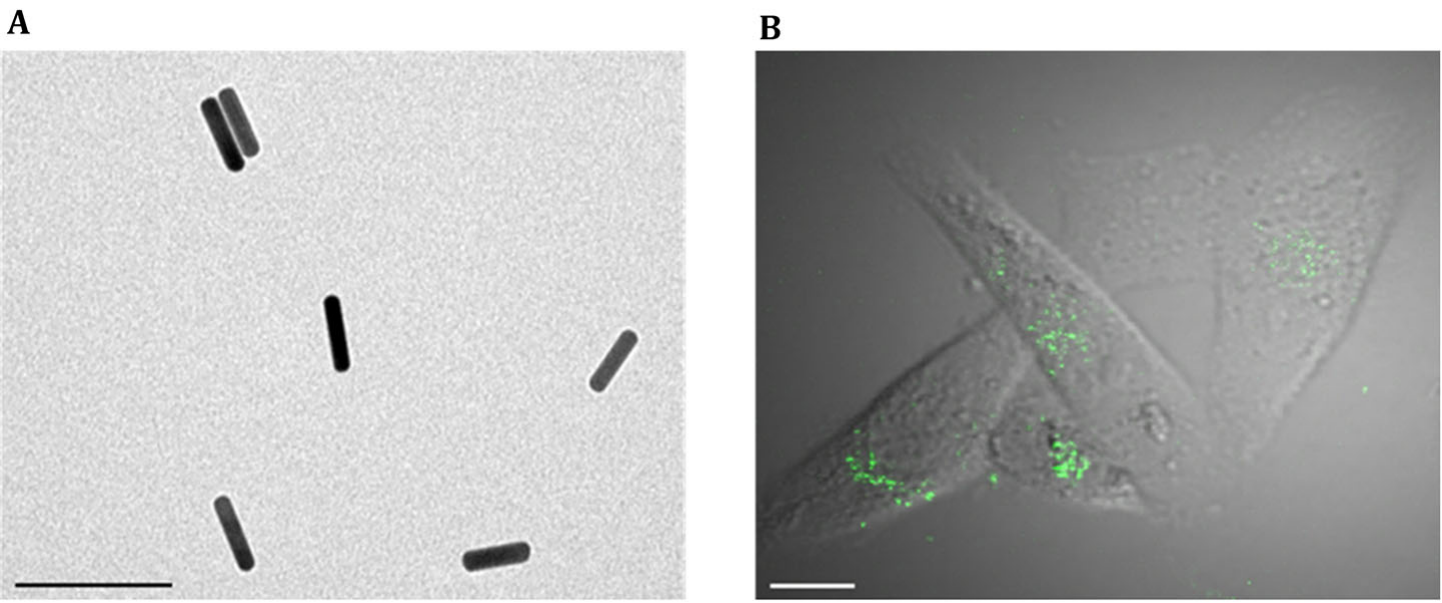
Observation of AuNR uptake into MDA-MB-231 cells. **A** TEM image displaying the size and shape of the AuNRs. The *scale bar* represents 100 nm. **B** MDA-MB-231 cells were incubated with 50 μmol/L AuNRs for 24 h, and the intracellular AuNRs were observed with a two-photon fluorescence confocal microscope. The *scale bar* represents 5 μm

AuNRs were prepared according to previously described procedures (Wang et al. 2011). The mean size of the AuNRs was calculated by measuring at least 100 particles using transmission electron microscopy (TEM). The AuNRs had an aspect ratio of 4.2 with a mean length of 51.2 ± 5.9 nm and a width of 12.2 ± 1.5 nm (Fig. 1A). The cellular uptake and trafficking of AuNRs are complex processes composed of multiple pathways of varying types (Zhang et al. 2013; Sahay et al. 2010; Doherty and McMahon 2009). To visualize the uptake of AuNRs by MDA-MB-231 cells, the cells were treated with 50 μmol/L AuNRs for 24 h and observed under a two-photon fluorescence confocal microscope. As shown in Fig. 1B, the AuNRs exhibited highly efficient two-photon-induced luminescence, which clearly indicated the intracellular location of AuNRs in the cells. AuNR exposure caused no significant effects on the cell viability, as measured by the MTT assay (data not shown).

### Construction of transcriptome profiles

To assess global transcriptome changes in the MDA-MB-231 cells treated with AuNRs, the cells were treated with 50 μmol/L AuNRs for 24 h or were not treated (control). Then, an mRNA of each sample was extracted, a cDNA library of each sample was constructed, and Illumina sequencing was carried out as described above. In total, 37,576,176 and 47,340,538 100 bp sequence reads for the untreated cells (UT) and AuNR-treated cells (AT), respectively, were generated by Illumina HiSeq™ 2000 sequencing. The mean quality values and statistical distributions of the reads obtained were calculated to analyze the average data quality. As shown in Table 1, more than 98% of the reads of each sample had a mean quality value of at least 21, which indicated that these data were reliable and that the following analyses could be performed.

**Table 1:** Average quality (AQ) summary of the total reads

Sample	UT_1	UT_2	AT_1	AT_2
Raw Data	18,788,088	18,788,088	23,670,269	23,670,269
AQ [26, 40]	17,959,523	17,387,591	22,618,388	21,945,018
%	95.59	92.55	95.56	92.71
AQ [21, 25]	585,577	861,362	726,630	1,066,704
%	3.12	4.58	3.07	4.51
AQ [11, 20]	241,779	523,325	323,542	640,775
%	1.29	2.79	1.37	2.71
AQ [0, 10]	1209	15,810	1709	17,772
%	0.01	0.08	0.01	0.08

Next, the low-quality reads were filtered, and the remaining reads were used for the additional mapping steps. In the UT library, 30,346,180 reads were mapped to the Human Ensembl database or exon junction database, of which 23,852,032 were focused on a unique region. In contrast, in the AT library, 38,140,036 reads were mapped to the Human Ensembl database or exon junction database, of which 30,002,220 were focused on a unique region. Both total mapping rates of the two libraries were approximately 80%. Reads mapping to the unique region were used to analyze the distribution of the transcripts. In the two libraries, we found that 60.38%–58.94% of the transcripts were located in an exon region, 10.04%–10.88% of the transcripts were in an intron region, and 0.28%–0.25% of the transcripts were in an exon-intron region (Table 2).

**Table 2:** Reads mapping summary

	UT	AT
Reads1	18,788,088	23,670,269
Reads1 mapped	15,343,841	19,286,241
%	81.67	81.48
Reads2	18,788,088	23,670,269
Reads2 mapped	15,002,339	18,853,795
%	79.85	79.65
Total unique mapped reads	23,852,032	30,002,220
%	63.48	63.38
Exon region reads	22,689,702	27,902,112
%	60.38	58.94
Intron region reads	3,772,049	5,149,780
%	10.04	10.88
Exon–intron region reads	104,075	118,719
%	0.28	0.25

**Fig. 2:**
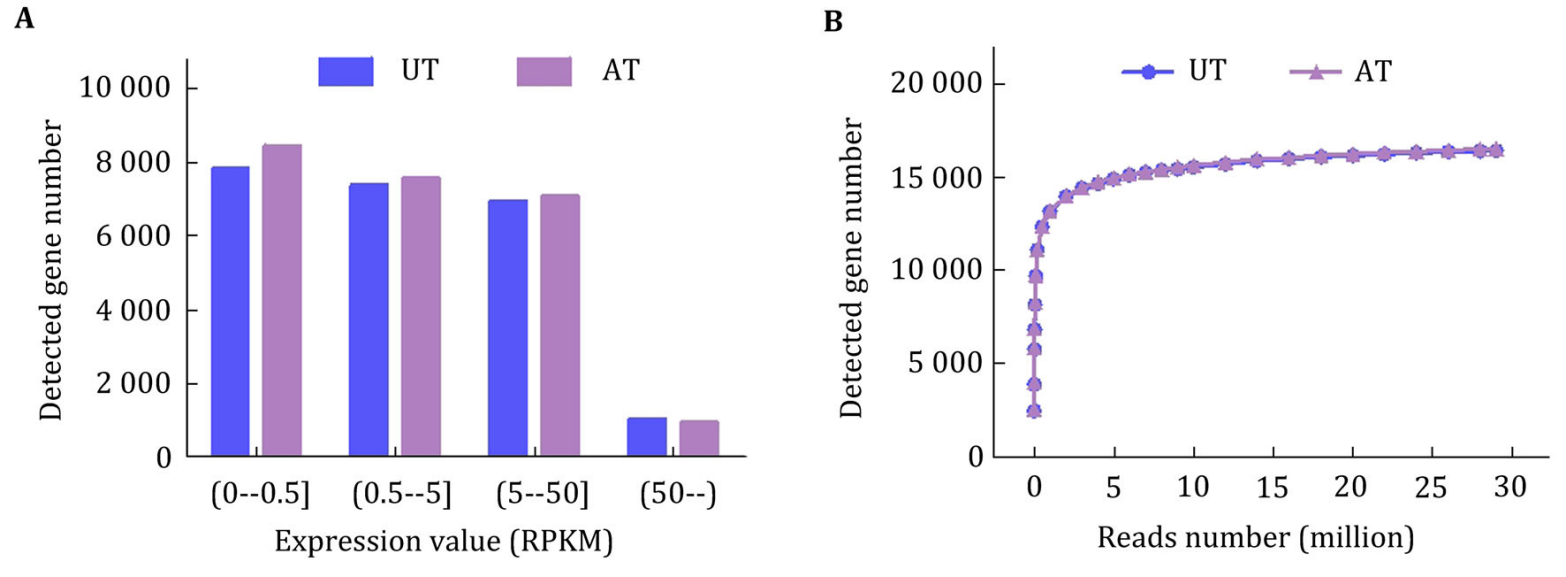
Summary of RPKM values of uniquely mapped genes and saturation analyses of the samples. **A** The RPKM value for each gene was calculated using uniquely mapped reads. **B** Each saturation *curve* was generated by randomly selecting a number of reads from each sample library and analyzing the number of genes detected. *UT* untreated MDA-MB-231 cells. *AT* MDA-MB-231 cells treated with 50 μmol/L AuNRs for 24 h

Furthermore, the gene expression level was quantified based on the number of reads that matched with exon regions. For both the UT and AT samples, approximately 35% of the identified transcripts had an RPKM value <0.5, 65% of the transcripts had an RPKM value more than 0.5, and 5% of the transcripts had an RPKM value >50 (Fig. 2A). Additionally, saturation curves were constructed for the two samples by subsampling the reads from each library and determining the number of genes detected. As shown in Fig. 2B, the curves of the two samples reached saturation at approximately 5 million sequenced reads at almost identical rates.

### Detection of differentially expressed genes

The DEGseq program was used to statistically analyze the differentially expressed genes between the two samples (Wang et al. 2010). In total, 6684 genes were found to be differentially expressed between the two samples, of which 3126 genes were up-regulated and 3558 genes were down-regulated in the AT sample compared to the UT sample. These data indicate that AuNRs may cause potential alterations to cellular functions by regulating mRNA transcript levels after entering MDA-MB-231 cells.

### Functional classification of differentially expressed genes

**Fig. 3:**
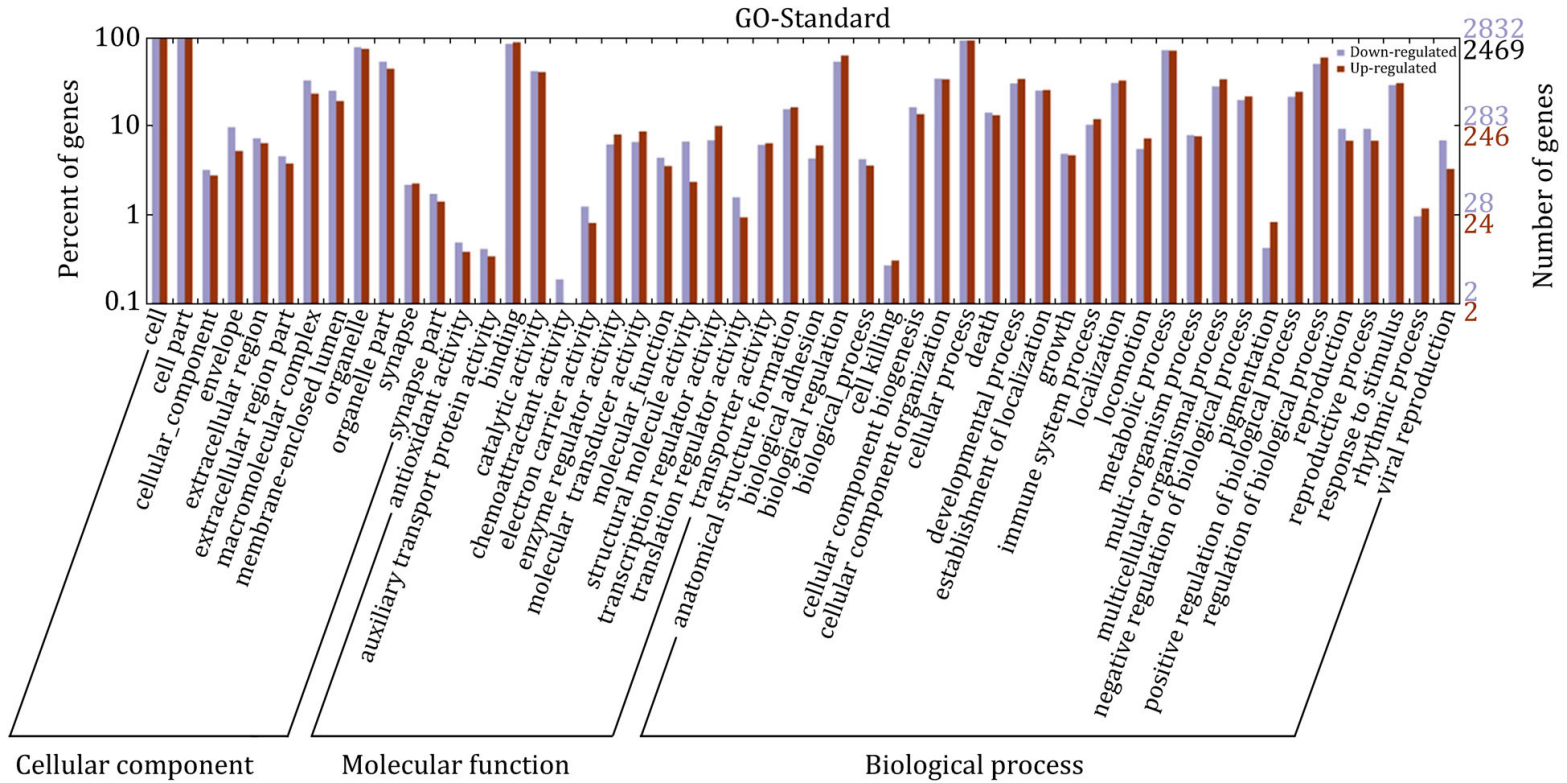
Functional categorization of differentially expressed genes. Gene Ontology (GO) terms at the 2nd level were plotted here. The *purple* and *red pillars* represent the down-regulated genes and up-regulated genes, respectively

The genes that were found to be differentially expressed between the two samples were categorized into Gene Ontology (GO) categories and fell into three major groups: cellular component, molecular function, and biological process. The abundant genes were categorized into 22 major functional groups (percentage of expressed genes >10) based on the GO categories. The top six functional categories included cell, organelle, binding, biological regulation, cellular process, and metabolic process (Fig. 3). The third level GO categories were consistent with these results and provided more information on the cellular functions and subcellular locations of these genes. The major functional groups based on these categories included intracellular part, intracellular organelle part, cytoplasmic part, protein binding, cellular catabolic process, cellular macromolecule localization, gene expression, protein transport, regulation of cell death, and cytoskeleton organization. In particular, the functional category of chemoattractant activity included differentially expressed genes that were largely down-regulated (Fig. 3). These analyses indicate that MDA-MB-231 cells exhibit a variety of responses to AuNRs and that their movement capability toward a chemoattractant signal might be specifically decreased by AuNR treatment.

### Variation of biological pathways in AuNR-treated MDA-MB-231 cells

The differentially expressed genes described above were also enriched for pathway analysis based on the Kyoto Encyclopedia of Genes and Genomes (KEGG) database. Multiple pathways were significantly impacted in AuNR-treated MDA-MB-231 cells, including Huntington’s disease, ribosome, spliceosome, pathways in cancer, RNA degradation, endocytosis, glycolysis/gluconeogenesis, apoptosis, insulin signaling pathway, regulation of actin cytoskeleton, etc. As energy production and the actin cytoskeleton are closely related to the chemoattractant activity of metastatic cancer cells, we further analyzed the glycolysis pathway and the regulation of the actin cytoskeleton pathway in AuNR-treated MDA-MB-231 cells.

**Fig. 4:**
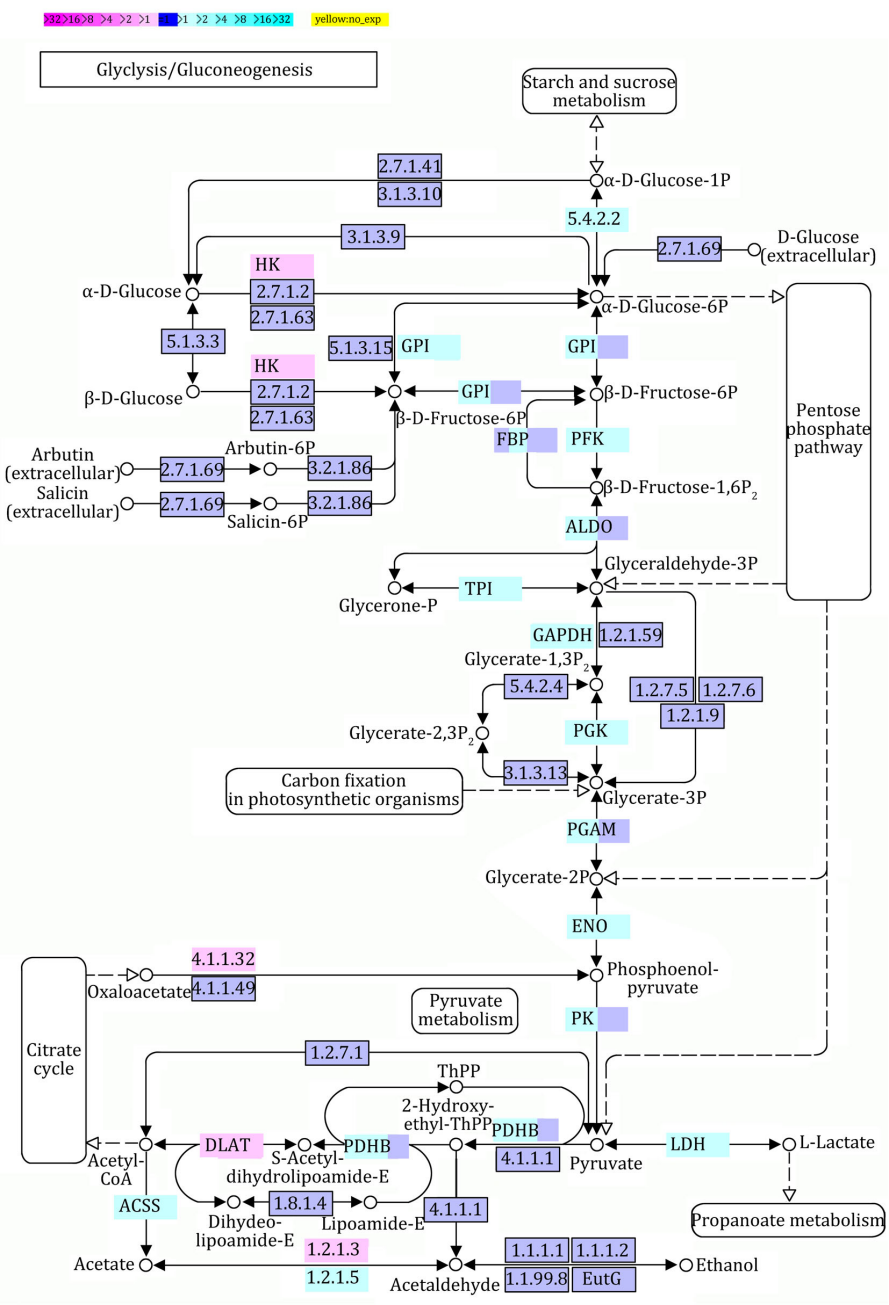
Differential gene expression in glycolysis/gluconeogenesis pathway in AuNR-treated MDA-MB-231 cells. *Magenta* and *cyan colors* represent up-regulated and down-regulated genes, respectively. The extent of regulation of the glycolysis/gluconeogenesis pathway provided by each gene is represented by *color grade*

Glycolysis is mainly an energy conversion pathway and takes place in the cytosol in eukaryotic cells. In glycolysis, the six-carbon sugar glucose is oxidized and split into two halves to create two molecules of pyruvate (3 carbons each). During glycolysis, the cell produces a relatively small amount of energy from glucose in the form of ATP. A net of only 2 ATP molecules are generated for each glucose molecule that starts down the glycolytic path. The pyruvate produced during glycolysis has three potential metabolic fates, to become acetyl-CoA, ethanol, or lactate. When oxygen is available, pyruvate can be converted to acetyl-CoA and enter the TCA cycle where it will be completely oxidized to produce ATP through oxidative phosphorylation. However, cancer cells often produce energy through glycolytic fermentation rather than oxidative phosphorylation. There are ten enzymes that catalyze the glycolytic steps that convert glucose into pyruvate. The activity of this pathway is regulated at key steps to ensure that glucose consumption and energy production match the needs of the cell. In mammals, the key regulatory enzyme is phosphofructokinase (PFK), which catalyzes the rate-limiting committed reaction of glycolysis. PFK is activated by AMP and inhibited by ATP; there are also other regulatory mechanisms that act on PFK. In the AuNR-treated MDA-MB-231 cells, most of the genes involved in glycolysis, including *pfk*, were down-regulated (Fig. 4). The down-regulation of those genes may slow glucose metabolism, reduce energy production, and result in further potential alterations to cellular functions, such as chemoattractant activity.

**Fig. 5:**
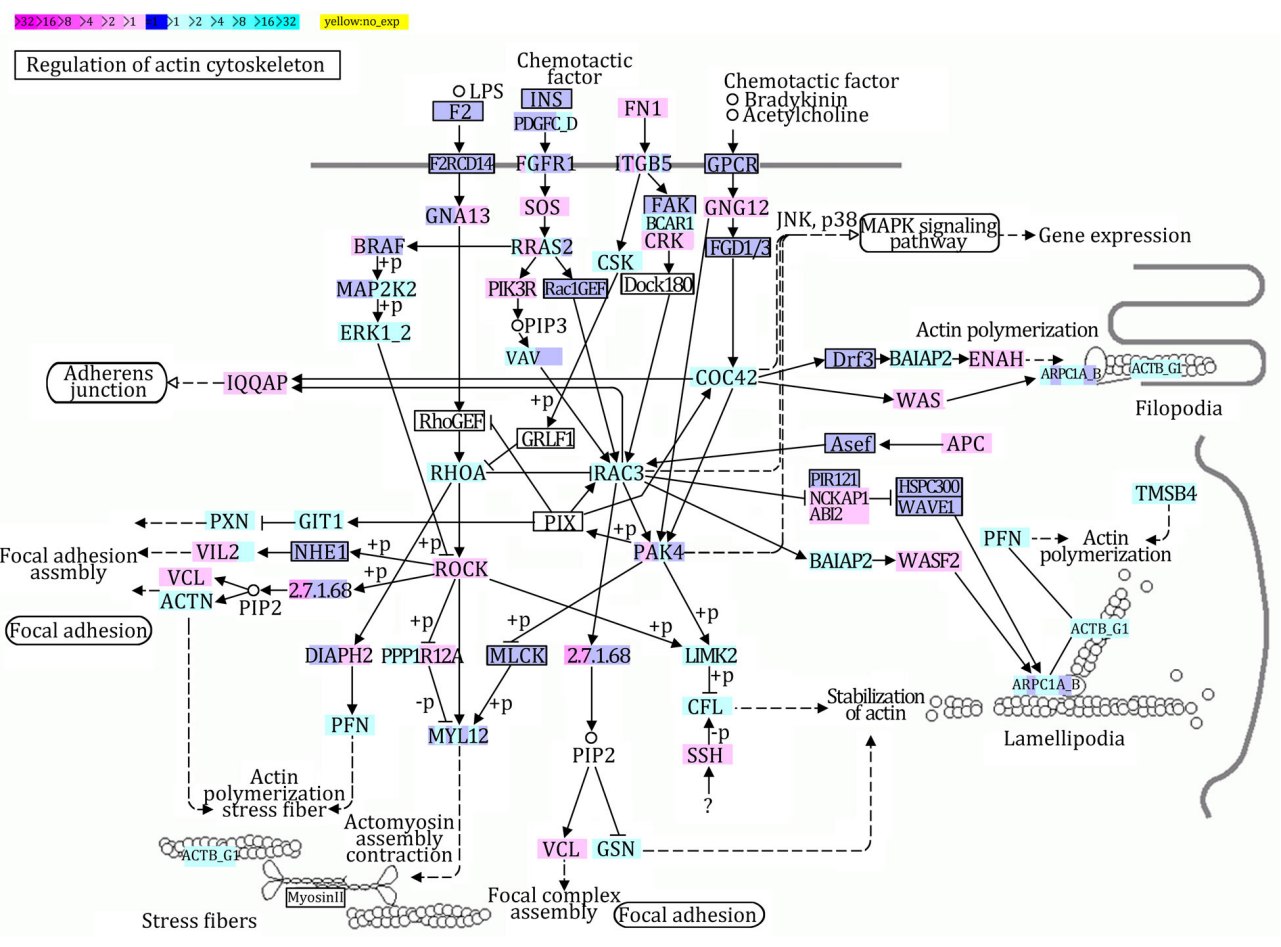
Different gene expressions in regulation of actin cytoskeleton pathway in AuNR-treated MDA-MB-231 cells. *Magenta* and *cyan colors* represent up-regulated and down-regulated genes, respectively. The extent of regulation of the regulation of actin cytoskeleton pathway provided by each gene is represented by *color grade*

The actin cytoskeleton mediates a variety of important biological functions in eukaryotic cells. In addition to providing a structural framework around which cell shape and polarity are defined, its dynamic properties provide the driving force for cell mobility, which is particularly essential for cancer cell migration and metastasis. The dynamic organization of the actin cytoskeleton is mainly regulated by small GTPases of the Rho family, in particular, RhoA, Rac, and CDC42, and requires an energy supply. Cells receive a variety of extracellular stimuli: soluble molecules (growth factors, hormones, and cytokines) that interact with cell surface receptors; adhesive interactions with the extracellular matrix; and cell–cell adhesions. These stimuli primarily regulate the actin cytoskeleton through Rho proteins. Once activated, Rho GTPases bind to a variety of effectors, including protein kinases and some actin-binding proteins. These complexes then directly or indirectly affect the local assembly or disassembly of filamentous (F)-actin. In AuNR-treated MDA-MB-231 cells, three major members of the Rho family (RhoA, Rac, and CDC42) were down-regulated (Fig. 5), and the down-regulation of these Rho family members may have impaired the assembly of the actin cytoskeleton and related cellular functions, such as chemoattractant activity.

## Conclusion

In this study, we investigated the mRNA transcript level changes of MDA-MB-231 human breast cancer cells treated with AuNRs employing RNA-seq technology. We observed the uptake of AuNRs into MDA-MB-231 cells using two-photon laser confocal microscopy. Then, total mRNA from AuNR-treated and untreated MDA-MB-231 cells was extracted, and a cDNA library for each sample was constructed. After Illumina HiSeq™ 2000 sequencing, transcriptome profiles were constructed and biological analyses were performed. In total, 3126 genes were found to be up-regulated, and 3558 genes were found to be down-regulated in AuNR-treated MDA-MB-231 cells. These genes presumably take part in multiple biological pathways, including glycolysis and regulation of the actin cytoskeleton, and impact various cellular functions, including chemoattractant activity. These results provide comprehensive insights into the distinct gene expression profile of MDA-MB-231 cells treated with AuNRs and also provide a foundation for further investigation of the effects of AuNRs on MDA-MB-231 cells.

## Materials and methods

### Materials

AuNRs synthesized by the seed-mediated growth method (Wang et al. 2011) were generously provided by Prof. Xiaochun Wu (National Center for Nanoscience and Technology). Cell culture medium and fetal bovine serum (FBS) were purchased from Hyclone (Logan, UT, USA). Cell culture petri dishes were purchased from BD Biosciences (San Jose, CA, USA). Other reagents were manufactured in China and were of analytical grade.

### Cell culture

MDA-MB-231 human breast cancer cells obtained from American Type Culture Collection (ATCC; Manassas, VA, USA) were cultured in DMEM medium supplemented with 10% FBS, 100 U/mL penicillin, and 100 μg/mL streptomycin at 37 °C in a humidified incubator with 5% CO_2_.

### Observation of AuNRs in MDA-MB-231 cells using two-photon laser confocal imaging

The size and shape of the AuNRs were characterized using transmission electron microscopy (TEM, Tecnai Spirit). To observe AuNRs in MDA-MB-231 cells, MDA-MB-231 cells were cultured on 35-mm petri dishes for 24 h and treated with 50 μmol/L AuNRs for 24 h. The cells were then washed with PBS, and fresh complete medium was added. The cells were observed using laser scanning microscopy (Olympus BX61W1 with Fluoview FV1000 software, Japan) with an excitation at 815 nm using a Maitai two-photon laser (Spectra Physics, Bozeman, MT, USA), and emission signals were detected in the green color channel.

### Cell treatment for transcriptomic analysis

MDA-MB-231 cells were equally seeded in two 100-mm cell culture petri dishes. When the cultured cells reached approximately 65% confluence, one dish of cells was incubated with 50 μmol/L AuNRs at 37 °C for 24 h, while the other dish of cells was left untreated as a control.

### RNA extraction and library construction

For each sample, approximately 5 × 10^6^ cultured cells were used for the isolation of total RNA with Trizol Reagent (Life Technologies, Shanghai, China). After selective depletion of rRNA, high-quality mRNA was extracted from each sample with an Oligotex mRNA Midi Kit (Qiagen, Shanghai, China) following the manufacturer’s instructions. Subsequently, mRNA was fragmented using 10× fragmentation buffer (Ambion, Austin, TX, USA) and run over a G50 Sephadex column (USA Scientific, Ocala, FL, USA) to remove fragmentation ions. The fragmented mRNA was randomly primed with hexamers and was reverse-transcribed using a Superscript II cDNA synthesis kit (Life Technologies). After second-strand synthesis, end-repair and ligation reactions were performed on the cDNA using paired-end adapters and amplification primers (Illumina, San Diego, CA, USA). The cDNA library was size-fractionated on a 2% TAE low-melt agarose gel (Lonza, Basel, Switzerland), and a slice of the cDNA lane at approximately 200 bp was selected to build clusters for the HiSeq™ 2000 sequencing system (Illumina) according to the manufacturer’s instructions.

### Reads mapping

After Illumina HiSeq™ 2000 sequencing with a read length of 100 bp, raw data from the sequencer were uploaded to the wapRNA (Zhao et al. 2011), which is a free web-based application for the processing of high-throughput RNA-Seq data from NGS platforms, such as Genome Analyzer of Illumina Inc. (Solexa). Low-quality reads (with an average quality value below 8) were filtered and the remaining reads were used for the additional mapping steps. The Human Ensembl database (version 69) was used as a reference database, and an exon junction database was created by the random combination of 92 bp sequences near the exonic junction points to annotate transcripts that originated from diverse exons due to alternative splicing. The BWA program (version 0.6.1-r104) was used to match-cleaned reads to the Human Ensembl database and the exon junction database for annotation successively, with the allowance of 5 mismatches for each read (Li and Durbin 2009).

### Quantification of gene expression level

The gene expression profiling analysis was based on the number of reads matching the exon regions. Genes that matched more than one read in the exon regions were considered to be expressed with hour statistical analysis method. Additionally, the expression value (RPKM, reads per kilo base of exon model per million mapped reads) for each gene was calculated to quantify their transcript levels using uniquely mapped reads (Mortazavi et al. 2008). Saturation curves were generated by randomly selecting a number of reads from each sample library and determining the number of genes detected.

### DEG detection

The DEGseq program was used to perform the statistical analysis of the differentiated gene expressions between the two samples (Wang et al. 2010). Genes with a *p* value <0.001 were considered to be distinctly expressed.

### Biological analyses

Functional classes were assigned according to GO mapping provided by the Ensembl database (Yi et al. 2006). The KEGG analysis was based on the results of the comparison between our mapped genes and the updated KEGG database (Kanehisa and Goto 2000).
